# Companion and free-ranging Bali dogs: Environmental links with personality traits in an endemic dog population of South East Asia

**DOI:** 10.1371/journal.pone.0197354

**Published:** 2018-06-05

**Authors:** Luca Corrieri, Marco Adda, Ádám Miklósi, Enikő Kubinyi

**Affiliations:** 1 Department of Ethology, Eötvös Loránd University, Budapest, Hungary; 2 Independent Researcher, AEC Anthrozoology Education and Canines, Naples, Italy; 3 MTA-ELTE Comparative Ethology Research Group, Budapest, Hungary; Memorial University of Newfoundland, CANADA

## Abstract

Dogs living on Bali Island have been free-ranging for thousands of years. A large group of expatriates sometimes adopt Bali dogs and keep them restricted to their houses and backyards, as is typical in modern western cultures. This provides us with the unique opportunity to compare the personality traits of dogs to their lifestyle either living as human companions or as free-ranging animals, exploring at the same time the impact of demographic variables (such as age, sex, and neutered status) on personality. After controlling for internal consistency of the scales and between-observer variation, we found that free-ranging Bali dogs were rated as less active, less excitable, less aggressive towards animals, and less inclined to chase animals or humans than Bali dogs living as human companions. Among free-ranging dogs, females were found to be more excitable. Females in the whole sample were also more fearful of people. The results of this preliminary study suggest that a change in lifestyle, i.e. being adopted, and living in a confined environment has negative consequences on some canine personality traits, such as activity/excitability, aggression towards animals, and prey drive.

## Introduction

**Fig 1:**
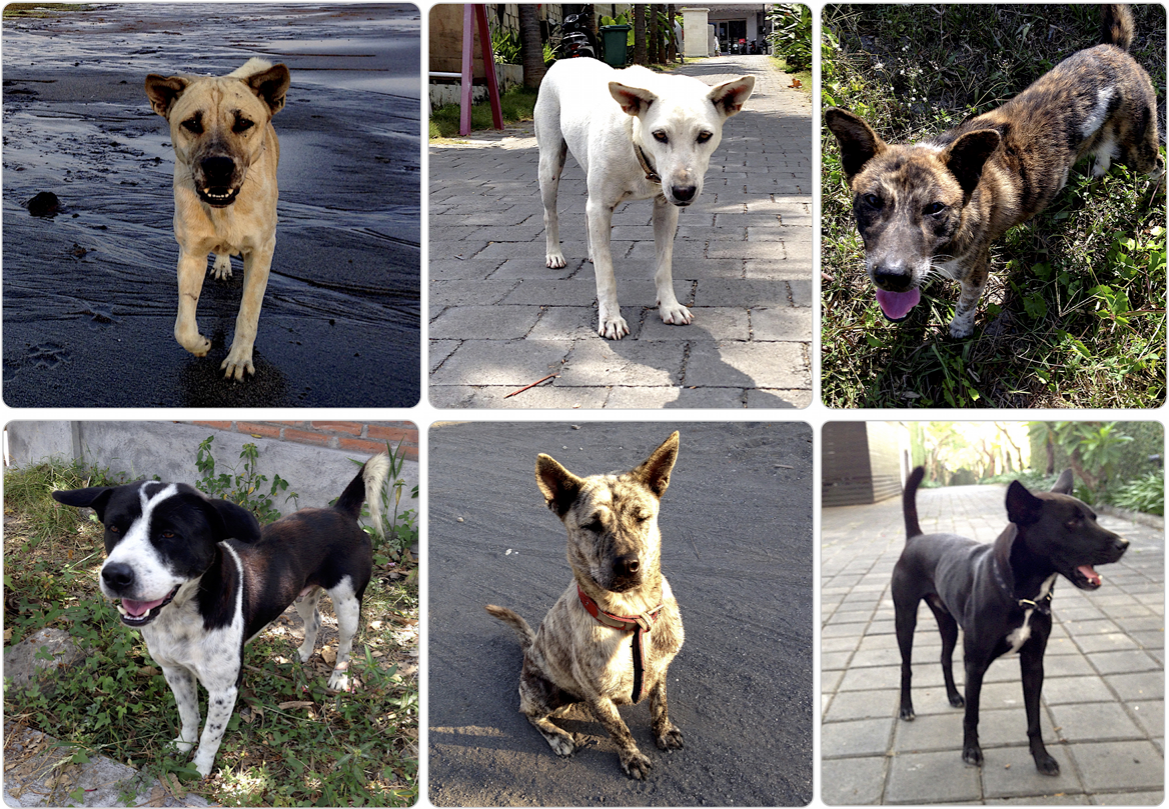
Bali dogs (Photo: Marco Adda).

“Bali Street Dogs” (also known as BSD or Bali dogs; Fig 1) represent an endemic dog population of Bali Island [1]. The population has critically dropped off in the last decade. While the Bali dog population was estimated to be between 600,000 and 800,000 in 2005 to 2008 [1,2], in 2008 an epidemic of rabies spread around Bali and the mass culling of dogs has ensued since then [3,4]. Due to that and to other major factors, the free-ranging dog population has been reduced by at least 25% since 2008, resulting in an estimated number of circa 150,000–160,000 dogs remaining today.

The Bali dogs are close relatives of ancient Asian breeds [5], like the Australian Dingo and the Chow Chow. A DNA analysis has shown that the history of this breed may date back to the human migration and colonization of South Indo-China, before the end of the last glaciation period [1]. At that time, Bali was connected with the continent by a bridge of land, which later submerged due to rises in sea levels [6]. Bali then became an island approximately 10,000 years ago, and the existing dogs, due to geographic isolation, differentiated into an endemic breed around at least 3,000 years ago [5]. In the remote past, humans visited Bali less frequently than the neighbouring Java Island, consequently limiting the influx of other dogs on Bali dogs. In addition, a strict rabies control program in effect since 1926 prohibited the introduction of other dogs to the island for most of the last century. Thus, the BSD population has likely free-bred and free-roamed for thousands of years with a limited genetic influx. Despite that, high levels of genetic diversity were found in microsatellite analyses [1]. Thus, geographic isolation was unlikely to be absolute and genetic diversity was probably enhanced at various times by the influx of new dogs.

The BSD has not been subjected to a domestication process as modern western dog breeds have been. They probably have not been deliberately selected for any particular phenotypical traits, including morphological or behavioural traits. However, Bali dogs are known for being skilful in guarding. Thus, although this behaviour occurs spontaneously and is not the consequence of human training, we may hypothesize that this trait has been unintentionally preferred across the centuries, and therefore has become a prevalent characteristic for BSDs.

Bali dogs have traditionally lived free around the streets and villages on the island, and were not restricted to houses or kept behind fences. They have lived alongside Balinese people and were welcomed or tolerated and integrated into all the daily activities of life on Bali. Balinese have historically allowed dogs to free roam, even those they owned [7,8]. This has resulted in dogs living mostly in proximity to humans and yet they are very independent. Free-ranging dogs in Bali are typically scavengers. Their two main sources of food are represented by waste or the abundant ceremonial offerings that Balinese leave in numerous temples, along the streets, and outside their households.

While some dogs may associate with an individual human, a house or a group of people living in a certain area, others do not associate with anyone in particular. This scenario was also supported by the Balinese predominant religion of Hinduism and values rooted in its mythology [7], to which Balinese are devoted. However, since the second half of the twentieth century and particularly in the last decade, the general attitude of the Balinese toward Bali dogs has undergone a major transformation. Although there are still many cases of integration or tolerance, there are two new trends in the human-dog relationship. Some Balinese now establish a closer affiliative relationship with Bali dogs, treating them as family members, similarly to a typical modern western attitude towards pet dogs. In contrast, other Balinese treat these dogs with suspicion or aggression since they consider them carriers of disease or find them disruptive and annoying, especially in the areas designed for tourism.

Moreover, recently, a large group of expatriates mainly from Europe, US, Japan and Australia have moved to Bali, and sometimes adopt Bali dogs and restrict their movements to houses or backyards, as is typical in modern western cultures. This new living situation allows us to compare the behaviour of Bali dogs living as human companions to dogs that remain free-ranging.

Most of the research on dog personality targets purebreds and is mainly motivated by needs related to improving the success of adoption rates, predicting susceptibility towards behavioural problems, or suitability for service dogs or police dogs [9]. In contrast, free-ranging dogs’ personalities have rarely been observed, especially when referring to mongrels or endemic dogs in non-western countries. BSD offer a unique opportunity to study a genetically homogeneous population that has lived halfway between the feral wild environment and the domestic human environment. Thus, recent BSDs' lifestyle may be analogous to that of dogs living during the early stages of domestication [10].

Furthermore, there are remarkably few studies investigating the links between personality traits and intrinsic or extrinsic factors. For example, previous studies suggested that some personality traits change during aging. Thus boldness decreased with age in an Australian sample [11], calmness increased with age in a German pet dog population [12], and activity level dropped with age according to a meta-analysis and results from a US sample [13,14]. Fearfulness, aggression towards people, responsiveness to training and aggression towards animals increased with age (from 6 months to 26 months of age) in Border collies living in Austria [15]. Sex is related to personality traits, too; for example, females were found to be more sociable in a German sample [12] and more trainable in Italy [16]. Breed was also shown to influence personality traits like playfulness, curiosity/fearlessness, sociability and aggressiveness at least in dogs living in Sweden [17].

In this study, our aim was to analyse whether free-ranging Bali dogs and companion Bali dogs differ in personality traits. We hypothesized that lifestyle (i.e. living constrained under the supervision of humans) could affect some personality traits. Companion dogs gain experience of the environment only when they go out with their owners and usually are not free to explore it by themselves. This could affect activity and sociality as well, because companion dogs have less exposure to other dogs and the environment when compared to free-ranging dogs. In addition, we examined how sex, neutered status, age, and their interaction with lifestyle are related to personality traits.

## Materials and methods

### Study sites

**Fig 2:**
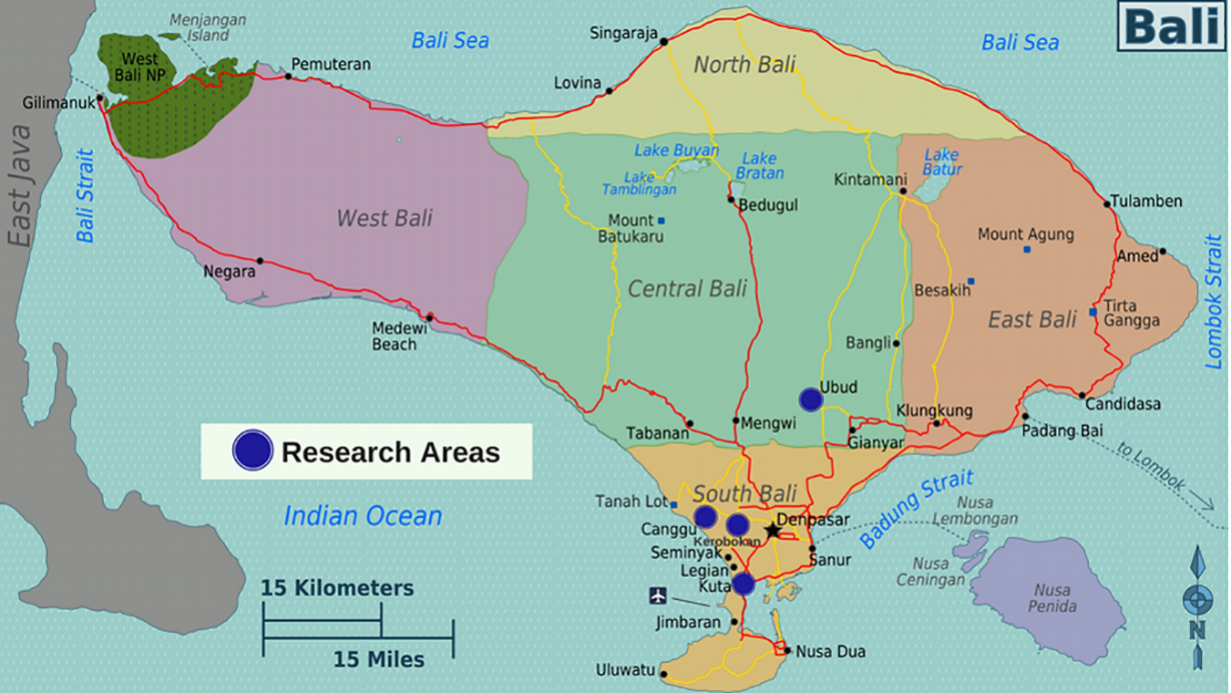
Map of Bali and research areas (Authors: Burmesedays, Peter Fitzgerald, Marco Adda [CC BY-SA 4.0–3.0–2.5–2.0–1.0]).

The study was conducted between March and November 2014 on Bali Island, after a preliminary period of two years of observations by Marco Adda, co-author of the study. Specific observations and the collection of questionnaire data started in March 2014, at the end of the rainy season (December-February). The research was constrained to a few areas (latitude, longitude): Ubud (-8.519268, 115.263298), in the region of Gianyar, and Canggu (-8.651221, 115.162236), Kerobokan (23.070508, 108.198500), and Kuta (-8.723796, 115.175228) in the region of Badung (Fig 2).

### Subjects

A total of 105 Bali dogs were surveyed using the 75 item “Dog Personality Questionnaire” (DPQ; Jones, 2008). Thirty dogs in the sample were less than 1 year old, and had to be excluded from the DPQ analysis, due to the fact that personality consistency was found to be stronger in adult dogs than in puppies [18]. In the adult sample (>1 year old), 15 dogs were companion animals and 60 dogs were free-ranging.

The questionnaires for companion dogs were filled out by their owners (N = 11 owners; 7 owners rated 1 dog, 4 owners rated 2 dogs). For free-ranging dogs the questionnaires were filled out by caretakers (N = 12; 4 caretakers rated 1 dog, 4 rated 2 dogs, 2 rated 3 dogs, 1 rated 4 dogs, and 1 caretaker, M.A., co-author of the study, rated 38 dogs, see *Behavioural observations* below). All owners and caretakers were expatriates.

For demographic data of the adult dogs, see Table 1. Neither age, nor sex ratio or neutered status ratio differed between the free-ranging and companion dog populations.

**Table 1:** Demographic data of the adult (>1-year-old) companion and free-ranging dog population.

Population	N	Age ± SD (months)	Age range (months)	Female %	Neutered %
Companion	15	32 ± 17	14–66	40%	80%
Free-ranging	60	33 ± 20	12–132	36.7%	60%

### Companion dogs

Live in the resident’s houses and enclosed gardens, and typically go out on a leash with an owner or a dog walker. Thus, these dogs are fully dependent on humans.

### Free-ranging dogs

May live fully free-ranging or they may have an owner or one or more caretakers. A caretaker does not recognize her/himself as an owner although s/he might take some care of the dog; provide some food, medical support, and occasionally shelter. This type of relationship with humans is typical of free-ranging dogs and in particular, village dogs. Consequently, these dogs are not restricted and are left free to roam, which allows the dogs a degree of independence from their owners or caretaker in many regards, including food provision. Most refer to these types of dogs as village dogs [19–21].

A preliminary analysis, using the DPQ, conducted on the adult sample to determine whether free-ranging (N = 38) and dogs that clearly associate with a house and yet are not restricted (N = 22) differed in personality traits showed no difference between them. Hence, we combined these two groups of dogs into the single category “free-ranging”. Importantly, feral dogs were not included in this study, as they display a strong continuous avoidance of human contact [22,23]. In Bali, feral dogs are represented by only a small minority of animals.

### Behavioural observations

The familiarity of the caretakers with the free-ranging dogs had two origins: the dogs roamed free in the area where the caretaker lived, or the caretaker visited the area where the free-ranging dogs lived in order to take care of them. The caretakers who filled out the forms were people with relevant experience with dogs in general, and with Bali dogs, in particular; they occasionally provided them with food and other care. All of the caretakers worked for associations or organizations involved in dog welfare, although they participated in the study independently from these associations. M.A., who filled in questionnaires for 38 free-ranging dogs (see *Subjects*), observed the subject dogs on a daily basis over a two-year period before filling out the questionnaires. He conducted preliminary observations on approximately 4000 dogs from 2012–2014 in different areas of the Island of Bali (including areas with both high and low distributions, Fig 2). In the periods March-June and September-November 2014, observations of free-ranging dogs were restricted to specific areas, such as Ubud and Canggu, and carried out on individuals that were identified as suitable for the study. The “suitability” of areas and dogs was based upon the recurring presence of dogs within a certain area, which allowed a wider range of observations and a more effective evaluation of their behaviour. M.A. invested about 5 hours per day observing the free-ranging dogs included in the study, usually 2 hours in the morning (between 7 am and 10 am), 2 hours at sunset (5–7 pm), and 1 hour at night. More attention was given to nocturnal observations during those phases when dogs increased their nocturnal activities. Considering an average period of 26 weeks of observations, 5 hours per day, we estimate M.A. undertook approximately 900 hours of specific observations during the study.

Dogs were observed while performing a wide variety of behaviours and activities such as scavenging, feeding, sleeping, travelling, and participating in intra-specific and extra-specific interactions such as socializing and playing. Observations included handwritten notes, pictures and video (device iPhone 5).

### Age estimation

For 22 free-ranging adult dogs with a caretaker and for all companion dogs (N = 15) we recorded the dogs’ age in months as was provided by the people who knew the dog(s). For 24 free-ranging dogs, in certain cases, it was possible to ask animal welfare practitioners active in those areas where the dog lived, or residents of the area, if they knew the dog’s approximate age. In other cases (N = 14), age was estimated by observing the teeth, hair, skin and coat of the individual.

### Questionnaire data

The Dog Personality Questionnaire (DPQ) contains 75 items in the long version used in this study [24]. Each item is rated on a 7-point scale ranging from 1 (disagree strongly) to 7 (agree strongly). We used the Scoring Key for the DPQ Long Form provided by the author (Jones, 2008), to calculate the factor and the facet scores; each factor has 2–4 facets (Table 2). We adapted some of the questions to apply to the free-ranging context. For example, item 11: “When off leash, dog comes immediately when called” assumes that being off leash was occasional and a non-ordinary condition, while for free-ranging dogs being off leash is a basic condition. Hence, we considered only the general recall for free-ranging dogs by omitting the “when off leash” from the question, and including only the statement “The dog comes immediately when called”. Another example is item 23: “Dog behaves aggressively when a person (e.g., visitor, delivery person) approaches the house or yard”. We considered the “house or yard” as the area where the free-ranging dog regularly visits, or the house with which s/he is usually associated.

**Table 2:** Factors, facets, item numbers, Cronbach’s alpha and inter-rater reliability of the Dog Personality Questionnaire (DPQ).

DPQ	Factors and facets	Item numbers	Cronbach’s alpha (N = 75)	ICC (N = 19)
**Factor 1**	Fearfulness*		**0.877**	**0.910**
Facet 1	Fear of People*	**R1, 12,** 30, **47,** 54	**0.749**	**0.910**
Facet 2	Nonsocial Fear	**6, R19,** 24, **R38,** R58	0.630	**0.814**
Facet 3	Fear of Dogs	R9, **21, 36,** 66, **70**	0.662	**0.839**
Facet 4	Fear of Handling	**28,** 32, 42, **61, 74**	0.520	**0.777**
**Factor 2**	**Aggression towards People**		**0.868**	**0.902**
Facet 1	General Aggression	**13,** 23, **R33, 68,** 73	**0.768**	**0.848**
Facet 2	Situational Aggression	2, 17, **43, 51, 62**	**0.854**	**0.916**
**Factor 3**	Activity/Excitability*		**0.867**	**0.928**
Facet 1	Excitability*	**27, 53,** 55, **R69**, 72	**0.824**	**0.797**
Facet 2	Playfulness	R3, **R16, 31,** 46, **59**	0.628	**0.815**
Facet 3	Active Engagement	**R10**, 14, **25, 40,** 48	0.632	**0.872**
Facet 4	Companionability*	7, **35, R44, 63,** 67	**0.725**	**0.928**
**Factor 4**	**Responsiveness to Training**		0.576	**0.928**
Facet 1	Trainability	37, R45, **R50, R64, 71**	0.504	**0.872**
Facet 2	Controllability	R4, **11, R18,** R29, **56**	0.460	**0.819**
**Factor 5**	Aggression towards Animals*		**0.817**	**0.860**
Facet 1	Aggression towards Dogs	**5,** 8, **R34,** 57, **R60**	**0.743**	**0.921**
Facet 2	Prey Drive*	**15,** 22, 26, **39, 65**	**0.804**	**0.833**
Facet 3	Dominance over Other Dogs	**20,** 41, **R49,** 52, **75**	0.531	**0.769**

An R in front of an item indicates that the item is reverse coded. High internal consistencies (Cronbach’s alpha above 0.7), and high inter-rater reliabilities (ICC above 0.7) are bolded.

Asterisk (*) marks factors and facets that were included in the final DPQ analysis due to high internal consistency and inter-rater reliability (see text for more details).

### Data collection and statistics

The questionnaires were filled out online, and none of the entries were incomplete. The individual scores from the questionnaire were calculated by using the factor structure from [24] (Table 2). The scores of the factors were calculated by taking the mean of all the facet scores that belong to that factor. Cronbach’s alpha was calculated for each factor and facet in order to verify the reliability of the data. We excluded factors and facets with Cronbach’s alpha < 0.7 from further analysis (Nonsocial Fear facet, Fear of Dogs facet, Fear of Handling facet, Playfulness facet, Active Engagement facet, Responsiveness to Training factor, Trainability facet, Controllability facet, and Dominance over Other Dogs facet, see Table 2), because these factors and facets did not represent stable underlying traits applicable across each dog population.

Since M.A. provided 38 assessments for adult free-ranging dogs, non-parametric median comparison tests (Mann-Whitney U tests) were used to determine whether there was evidence of consistent between-observer variation that could have led to bias in rating, similarly to [25]. For example, the median of Fearfulness for all dogs rated by observer M.A. was compared to the median ratings from each other free-ranging dogs’ observer. Significant differences (p < 0.05) emerged for Aggression Towards People factor, General Aggression facet, Situational Aggression facet, and Aggression Towards Dogs facet which indicated consistent between-observer variation. Therefore, these traits were excluded from further analysis.

In addition, 19 dogs were assessed by both M.A. and other observers. Data were analysed using intra-class correlations (ICC, average measure for absolute agreement, 2-way random model) to establish the inter-rater reliability. We found robust reliability for each of the facets and factors (ICC > 0.7–0.9, Table 2).

To investigate the relationship between the personality traits and the independent variables, we carried out regression tree (also known as decision tree) analyses, one for each of the remaining factors and facets (displayed in Table 2 marked with asterisks). Age, lifestyle (free-ranging/companion), sex, and neuter status were used as independent variables. Regression trees are ideal for analysing complex numeric and/or categorical data and detecting non-linear relationships [12]. We decided to use this method, because the relatively large number of explanatory variables used in this study does not facilitate the revealing of complex interactions when utilising more popular univariate analyses. We used the CHAID (Chi-Squared Automatic Interaction Detection) statistical method. CHAID uses an F test if the variable is continuous (e.g. the dog’s age) and χ^2^ if the variable is categorical (e.g. sex). CHAID chooses the independent variable that has the strongest interaction with the dependent variable. Categories of each predictor are merged if they are not significantly different with respect to the dependent variable. The output is a tree diagram with a parent node at the top containing the entire data set and mutually exclusive child nodes. In one node, individuals have similar values for the dependent variable. The number of data divisions is determined using a cross-validation procedure by randomly drawing samples from the data set to evaluate the predictive error of the tree. We specified the minimum number of cases as 30 for parent nodes and 15 for child nodes. SPSS 22.0 was used for all analysis.

## Results

The factors Fearfulness, Activity/Excitability, and Aggression Towards Animals and the facets Fear of People, Excitability, and Prey Drive (marked with asterisk in Table 2) were analysed by utilising regression trees.

In the case of the Fearfulness factor, the independent variables had no effects on the scores.

**Fig 3:**
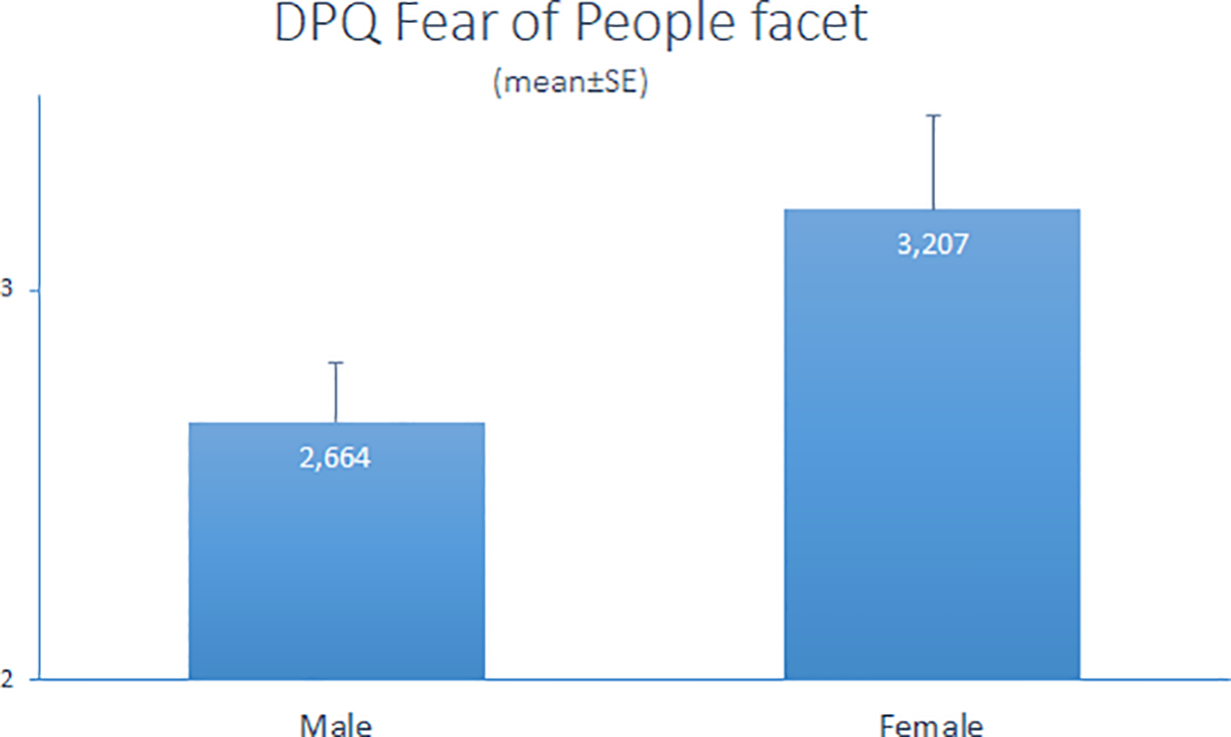
Female Bali dogs had higher scores in the DPQ Fear of People facet than males (p<0.05).

Sex affected the Fear of People facet, females had higher scores (F_1,73_ = 4.041, p < 0.05, Fig 3).

**Fig 4:**
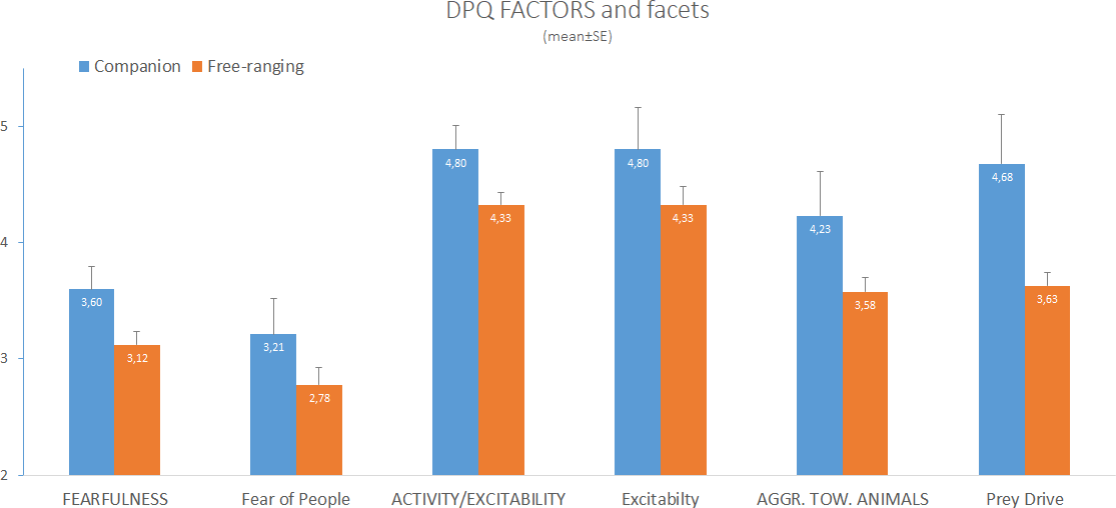
Comparison of companion and free-ranging Bali dogs in DPQ factors and facets. Factors are in capital letters. Asterisk (*) indicates significant differences (p<0.05).

Free-ranging dogs had lower scores on the Activity/Excitability factor (F_1,73_ = 4.238, p < 0.05), Excitability facet (F_1,73_ = 12.387, p = 0.001), Aggression towards Animals factor (F_1,73_ = 8.317, p < 0.01), and Prey Drive facet (F_1,73_ = 11.059, p = 0.001, Fig 4).

Finally, we detected an interaction between lifestyle and sex: among free-ranging dogs females had higher scores on the Excitability facet (F_1,58_ = 4.410, p < 0.05).

## Discussion

Free-ranging dogs represent nearly 80% of the world’s dog population, however studies detailing their behaviour and personality are currently lacking [26]. Free-ranging dogs, in general, can be socialized to humans, can build trust based on affection [27], and adopted street dogs can become companions. However, we hypothesise that free-ranging and pet population dogs differ in their behaviour as a consequence of their different life experience.

Although the results should be regarded as preliminary (due to the low sample size of companion dogs), when we compared the personality of adult free-ranging Bali dogs living as scavengers and adopted Bali dogs living as companions, we found that free-ranging dogs were scored as less active/excitable, less aggressive towards animals (including dogs), and less inclined to chase animals and people than companion dogs. According to the observers’ judgments, Fearfulness was similar in free-ranging and companion dogs.

The divergence in personality traits between the free-ranging and companion populations could come about due to differential survival rates in the two populations, e.g. aggressive free-ranging dogs may die earlier due to injuries. Alternatively, the populations may be exposed to different environmental influences, e.g. companion dogs may show increased aggression toward animals, because of the limited experience with them, due to the more restricted environment (living in a house or garden, occasional walks on leash). Given the nature of the present study, we were not able to reveal any effects of differential selection processes, so below we summarise only the possible environmental factors, which may have an effect on either free-ranging dogs or companion dogs.

Free-ranging dogs could generally behave more calmly, due to a confidence derived from their continuous experience of the street environment, cars and traffic, and exposure to other dogs, animals, and humans. Tiira and Lohi [28] found that increased fear in older family dogs might be the result of reduced experience of the surrounding non-social and social environment. Bali is a challenging environment for free-ranging dogs (e.g. because of car accidents, poisoning, periodic culling, snake bites), therefore, owners may prefer to confine their dogs in or around the house. In addition, higher control of the dogs’ behaviour including some punishment, may also contribute to increased activity, excitability, and aggression in companion dogs. In contrast, free-ranging dogs do not undergo similar types of behavioural control by humans during their development. Chasing cats, dogs, or people is certainly costly due to the chance of injury or death. Considering that in India 63% of the total mortality of free-ranging dogs is influenced by humans [29], free-ranging dogs with a high prey drive are probably selected out early in life by road accidents and culling. The selection process, in addition to the broader experience acquired, could explain why free-ranging Bali dogs showed less activity/excitability, aggression, and chasing behaviour.

Free-ranging dogs were perceived as less aggressive towards animals in general. Within stable social groups where the positions in the hierarchy are established, aggressive behaviour in free-ranging dogs tends to be limited [30]. Although a previous study about agonistic behaviour in free-ranging dogs in India has shown that overall, levels of aggression were higher among the adult females [31], in our study, we did not find that free-ranging female dogs were more aggressive towards other animals.

While lifestyle had no effect on the factor Fearfulness, we found that, in contrast to males, female dogs were more fearful of people. This result is supported by similar findings in a study by Kubinyi et al. [12]. The sex of the dog was the most predictive variable of boldness in a large sample of German companion dogs; females were found to be considerably less bold compared to males [12]. In the current study, female dogs were also found to be more excitable among free-ranging dogs. Since free-ranging dogs are scavengers, females raising puppies face a challenging environment with resource limitation [32], which might lead to elevated level of excitability in general. However, a significant proportion of females were neutered, so other, unidentified factors also likely influenced this behaviour.

One of the limitations of this research is that similarly to other questionnaire studies, the different attitude of the respondents might have biased the results. We can assume for example, that the owners of companion dogs were more concerned about their dogs than the observers of the free-ranging dogs, or owners perceived their dog as more active/excitable simply because they were confined to a limited area with their dogs for extended periods of time. Although the raters of free-ranging dogs spent considerable amounts of time observing individuals, we cannot assume that there was no systematic bias in the ratings of the companion and free-ranging dogs.

One virtue of this study is the relatively insular population. In the past, artificial selection for morphology or behaviour has not been exerted on Bali dogs, in contrast to the Western dog population. The fact that in recent years some of the Bali dogs have been adopted by expatriates and live a “Western” lifestyle, allowed us to uncover a few potential differences between the personality of free-ranging and companion dogs, without the confounding effects of breed differences. It is possible, although further investigations should confirm, that a change in lifestyle, i.e. being adopted, and living in a confined environment has negative consequences on some canine personality traits, especially on activity/excitability, aggression toward animals, and prey drive, and the less challenging experience of confined living relaxes the behaviour of companion dogs in comparison to free-ranging individuals.

## Supporting information

S1 File Data set in xls format. (XLSX)
